# Berberine promotes M2 macrophage polarisation through the IL-4-STAT6 signalling pathway in ulcerative colitis treatment

**DOI:** 10.1016/j.heliyon.2023.e14176

**Published:** 2023-03-01

**Authors:** Kai Xiong, Jia Deng, Tinghui Yue, Wenting Hu, Xinglin Zeng, Tao Yang, Tianbao Xiao

**Affiliations:** a Colorectal and Anal Surgery, The First Affiliated Hospital of Guizhou University of Traditional Chinese Medicine, No 71 Baoshan North Road, Guiyang, 550001, China; b Colorectal and Anal Surgery, Chengdu Anorectal Hospital, Chengdu, 610075, China

**Keywords:** Ulcerative colitis, Berberine, Inflammation, Macrophages

## Abstract

**Aim:**

This study focusses on the anti-inflammatory and immune-modulatory roles of berberine (BBR) in ulcerative colitis (UC) treatment. Additionally, the underlying mechanisms of BBR were systematically explored.

**Methods:**

A 3% (*w/v*) dextran sodium sulphate (DSS) solution was used for establishing the mice UC model. M2 macrophage polarisation was induced in RAW 264.7 cells using interleukin 4 (IL-4), whereas M1 macrophage polarisation was induced using lipopolysaccharide. Colon length, colon mucosa damage index (CMDI), and haematoxylin–eosin (HE) staining were used to evaluate colon damage induced by DSS. M1/M2 macrophages in the colon tissue were identified using immunofluorescence (IF) staining with CD86^+^ or CD163+. M1/M2 macrophages in the abdomen were examined using flow cytometry. An enzyme-linked immunosorbent assay was conducted to identify M1/M2 macrophage supernatant biomarkers in RAW 264.7 cells. Western blotting, immunohistochemical staining, and real-time PCR were performed to investigate the potential mechanisms of BBR for treating UC *in vivo and in vitro.*

**Results:**

BBR was found to prolong colon length, ameliorate CMDI and alleviate the colon's pathological changes in UC mice. In DSS-induced UC mice, M1 macrophages predominated. BBR promoted M2 macrophages and suppressed M1 macrophages in the colon and abdomen of DSS-induced UC mice. Additionally, BBR significantly decreased M1-specific markers (IFN-γ and IL-1β) while increasing M2-specific markers (IL-10 and TGF-β) in the supernatants of RAW 264.7 cells. BBR upregulated the mRNA expression of IL-4, STAT6, and Chil3 while downregulating TNF-α, IFN-γ, and NOS2 expression *in vivo*. Moreover, BBR activated the downstream targets of the IL-4-STAT6 signalling pathway and enhanced the phosphorylation of STAT6 *in vivo* and *in vitro* to polarise M2 macrophage.

**Conclusion:**

In UC mice, BBR suppressed M1 macrophages while promoting M2 macrophages. M1 macrophage suppression and M2 macrophage activation were strongly correlated with the anti-inflammatory and immune-modulating activities of BBR. BBR induced the polarisation of M2 macrophages by activating the IL-4-STAT6 signalling pathway, which contributed to its therapeutic efficacy against UC.

## 1 Introduction

Ulcerative colitis (UC) is a colon and rectum-involved inflammatory bowel disease with an unknown aetiology. Over the years, increasing global prevalence and fatality rates of UC have caused concern. Typical clinical manifestations of UC included bowel urgency, stomach discomfort, bloody diarrhoea, and tenesmus [1]. UC drastically decreases the quality of life and even leads to death owing to untimely treatment [2]. Besides, the available means fail to completely cure UC [3]. Usually, UC patients with mild-to-moderate symptoms are managed with anti-inflammatory therapies (e.g., 5-aminosalicylates or corticosteroids), and UC patients with severe symptoms are treated with immunosuppressants or anti-tumour necrosis factor (TNF) therapies. However, the complete remission of symptoms and improvement in QOL is not achieved in many patients [4]. Therefore, current therapeutic regimes are clinically inadequate and hence require more effective drugs or therapies for efficient UC treatment.

The basic pathology of UC is distensible inflammation in the colonic mucosa [5,6]. In the initial stage of inflammation, macrophages, which represent a crucial innate immunity population that regulates inflammation, lymphocyte activation and host defences, were recruited to the colonic mucosa for carrying out timely immune responses [7]. Two well-known outcomes of macrophage polarisation have been established based on different stimuli: classical macrophage (M1) activation with proinflammatory activity and alternative macrophage (M2) activation with anti-inflammatory activity [8,9]. The M1 macrophage phenotype is hallmarked by increased production of MHC class II molecules and proinflammatory cytokines. M2 macrophages, on the other hand, demonstrated improved tissue-repair capability as well as elevated anti-inflammatory activity [10,11]. Recently studies have reported that the colonic mucosa of UC is infiltrated by inflammatory macrophage M1 [12,13]. Furthermore, increased M1 and decreased M2 macrophages are frequently observed in the inflammatory UC colon tissues, and the disproportion between M1 and M2 macrophages is strongly correlated with UC progression [14]. Therefore, the effective regulation of macrophage polarisation between M1 and M2 can be a potential therapeutic strategy in UC treatment.

Recent emphasis has been focused on the activation of M2 macrophages by Th2 cytokines, particularly in UC therapy. Interleukin 4 (IL-4), a Th2 cytokine, has been shown to significantly enhance the activity of prostaglandin E2, TNF-α, and IL-1β in human monocytes, which contributes to its anti-inflammatory activities [15]. IL-4 activates transcriptional macrophage repressors, signal transducer, and transcription 6 (STAT6) activators, which contribute to M2 macrophage polarisation via Janus kinase 1/3/STAT6 activation and phosphoinositide 3-kinase signalling pathways [16,17]. Furthermore, the phosphorylation of STAT6 has been reported to be crucial for Th2 cell phenotype with IL-4-dominant *in vivo* and is an essential transcriptional mediator of IL-4-dependent repression [18]. Moreover, it can be assumed that the IL-4–STAT6 signalling pathway induces M2 macrophage polarisation and alleviates inflammation in UC colonic mucosa.

**Fig. 1:**
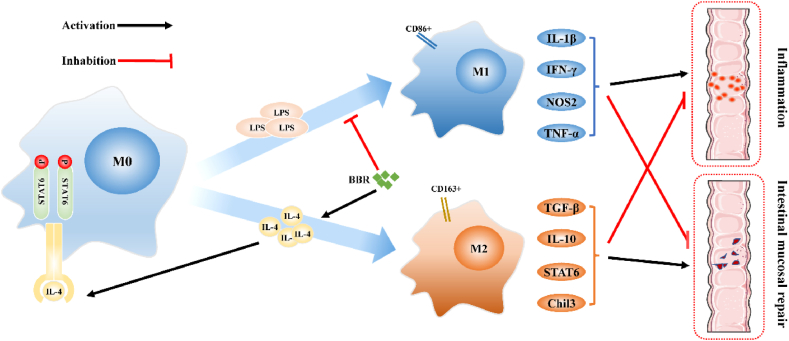
Schematic paragraph of BBR in treating UC.

In Asia, berberine (BBR), an isoquinoline alkaloid that occurs naturally, has been used extensively to treat gastrointestinal diseases. Numerous research has shown that BBR has anti-inflammatory, immune-modulatory, and anti-cancer activities [19,20]. However, the potential mechanisms of BBR against UC remain unknown. Therefore, this study investigated the therapeutic efficacy and possible mechanisms of BBR against UC, which helped to understand the therapeutic effects of BBR in treating UC (Fig. 1).

## 2 Materials and methods

### 2.1 Ethics statement

An ethics statement was previously provided [21]. IACUC-2018-010 was the approval ID.

### 2.2 Reagents

The detailed information of reagents applied in this study was listed in supplement 1.

### 2.3 DSS-induced UC mice

We referred to the procedures of Yang et al. [21], Cui et al. [22] and Li et al. [23] to develop 3% DSS-induced UC mice models. Fifty male Sprague Dawley (SD) C57BL/6 mice (Beijing Sibeifu Animal Breeding Center, China), weighing 20 ± 2 g, were randomly classified into five groups (each containing 10 mice): (1) Control group; (2) UC group; (3) mesalazine group (mesalazine, 0.364 g/kg·d^−1^); (4) low-dose BBR group (BBR, 20 mg/kg·d^−1^); (5) high-dose BBR group (BBR, 40 mg/kg·d^−1^). The control group drank water continuously for 14 days. Other groups received mesalazine or BBR for 7 days after 3% DSS (*w/v*) treatment. Stool samples, blood samples, and colon tissues were harvested and preserved at −80 °C.

### 2.4 Cell culture

Murine-derived Mφs RAW 264.7 cells (IBMS, CAMS/PUMC, Beijing, China) were cultured in DMEM with 10% foetal bovine serum and 1% penicillin/streptomycin at 37 °C in a 5% CO2 humidified environment. RAW 264.7 cells were treated with LPS (200 ng/mL) or BBR and IL-4 (20 ng/mL).

### 2.5 Colon length measurement and colon mucosa damage index (CMDI) evaluation

The entire colon (from the ileocecal to the anus) was obtained and measured using a ruler (0–20 cm). Subsequently, the colon tissues were cut along the longitudinal axis, and macroscopic histological changes in the colon were observed and recorded for CMDI evaluation. CMDI values were calculated as the degree of hyperaemia, oedema, erosion, and ulcer in the colon [24].

### 2.6 Staining of the colon tissue with haematoxylin-eosin (HE)

The whole colon was dissected and then flushed with ice-cold PBS. The following procedures were followed to create tissue samples from colon tissue: Paraformaldehyde fixation, xylene clearing, paraffin embedding, and microtome cutting into 6-mm-thick sections. Subsequently, the samples were stained with HE. Using photography and light microscopy at 200× magnification, the injury to the colon mucosa was determined histopathologically.

### 2.7 Identification of M1/M2 macrophages in the colon tissue

The immunofluorescence (IF) staining protocol of the colon tissue reported by Im K et al. [25] was followed. Briefly, the harvested colon sections were deparaffinised and rehydrated with 10% donkey serum (for primary antibody originating from goat) being used for immunofluorescence blocking. Fluorescent markers used for M1 and M2 macrophages were CD86^+^ and CD163+, respectively. TSA-FITC or CY3-conjugated secondary antibody and DPAI were used for visualization after treatment with the primary antibodies CD86^+^ (1:50) and CD163+ (1:50). Images were captured and obtained using fluorescence microscopy.

### 2.8 Examination of M1/M2 macrophages in the abdomen

Macrophages in the abdomen were examined using flow cytometry (FCM). Peritoneal M1 and M2 macrophages were identified as CD11b + F4/80+ CD86^+^ and CD11b + F4/80+ CD206+, respectively. Peritoneal macrophages from the abdomen of mice were collected in flow tubes and centrifuged for 5 min at a rate of 1000 rpm, where the supernatant was discarded. Thereafter, 100-μL MEDIUM A, 100-μL MEDIUM B solution, 100-μLpre-cooled flow staining buffer, 5-μL anti-human/mouse CD11b (M1/70), anti-mouse F4/80 antigen (BM8.1), 5-μL anti-mouse CD86 (B7-2) (GL-1) and 5-μL anti-mouse CD206 (MMR) antibodies were added to the flow tubes. For 20 min, all antibodies were incubated at room temperature and in the dark. Following this, 400-μL FCM staining buffer was used to resuspend the cells for FCM analysis.

### 2.9 Measurement of specific supernatant biomarkers in M1/M2 macrophages

Following the manufacturer's instructions, ELISA kits (Applied JYM Biotechnology, Beijing, China) were used to quantify the levels of IFN-γ, IL-10, IL-1β, and TGF-β in the serum.

### 2.10 Real-time reverse transcription PCR (qRT-PCR) for mRNA expressions

TRIzol reagent (Nordic Bioscience, Beijing, China) was used to extract total RNA from the colon tissues, which was subsequently used in a reverse transcription kit (Promega, Madison, USA) to produce cDNA. Table 1 lists the primer sequences used. SYBR Green PCR Master Mix and a 7500 fast real-time PCR system (Applied Biosystems, USA) were used to perform qRT-PCR. In addition, 2^−ΔΔ^Ct was used to calculate the relative messenger RNA (mRNA) expression.

**Table 1:** Primers sequences of IL-4, STAT6, Chil3, TNF-α, IFN-γ and NOS_2_.

Genes	Primer	Sequences (5′- 3′)
IL-4	F	TACCAGGAGCCATATCCACGGATG
	R	TGTGGTGTTCTTCGTTGCTGTGAG
STAT6	F	TACGGCATCTCCTGGCTGACTG
	R	GACGCTGGACTGTGGCAGAAAG
Chil3	F	GCCCACCAGGAAAGTACACAGATG
	R	GACCTCAGTGGCTCCTTCATTCAG
TNF-α	F	CGCTCTTCTGTCTACTGAACTTCGG
	R	GTGGTTTGTGAGTGTGAGGGTCTG
IFN-γ	F	GCACAGTCATTGAAAGCCTAGAAAGTC
	R	GCCAGTTCCTCCAGATATCCAAGAAG
NOS2	F	GCCCACCAGGAAAGTACACAGATG
	R	GACCTCAGTGGCTCCTTCATTCAG
β-actin	F	TATGCTCTCCCTCACGCCATCC
	R	GTCACGCACGATTTCCCTCTCAG

### 2.11 Immunohistochemical (IHC) staining of the colon tissue

Polyclonal *anti*-IL-4 (1:200) and rabbit anti-STAT6 (1:200) antibodies were used to stain paraffin sections of previously prepared colon tissue. The section images were photographed with the aid of the NIS Elements Imaging Software Version 4.0 at a magnification of 200×.

### 2.12 Protein expression analysis using Western blotting (WB)

According to a previously published protocol [26], WB analyses of RAW 264.7 cells and colon tissues were carried out. *Anti*-IL-4 (1:1000), rabbit *anti*-BATF (1:1000), anti-STAT6 (1:1000), rabbit *anti*-GATA3 (1:1000), *anti*-IFN-γ (1:500), *anti*-phospho-STAT6 (p-STAT6, Tyr641, 1:800), rabbit anti-NOS2 (1:1000), and GAPDH monoclonal antibody (1:8000) were used in the WB experiment. Image version 1.8.0 was used to analyse the results.

### 2.13 Statistical analyses

Data are presented as $\bar{x}$ ± s and analysed using the SPSS software programme (version 21.0). For data analyses, one-way ANOVA followed by the least significant difference test was conducted. *P* < 0.05 indicated the significance level. The visualization of the results was achieved using R (version 4.0.4) and GraphPad Prism (version 8.01) software.

## 3 Results

### 3.1 BBR prolonged mice colon length and ameliorated CMDI

**Fig. 2:**
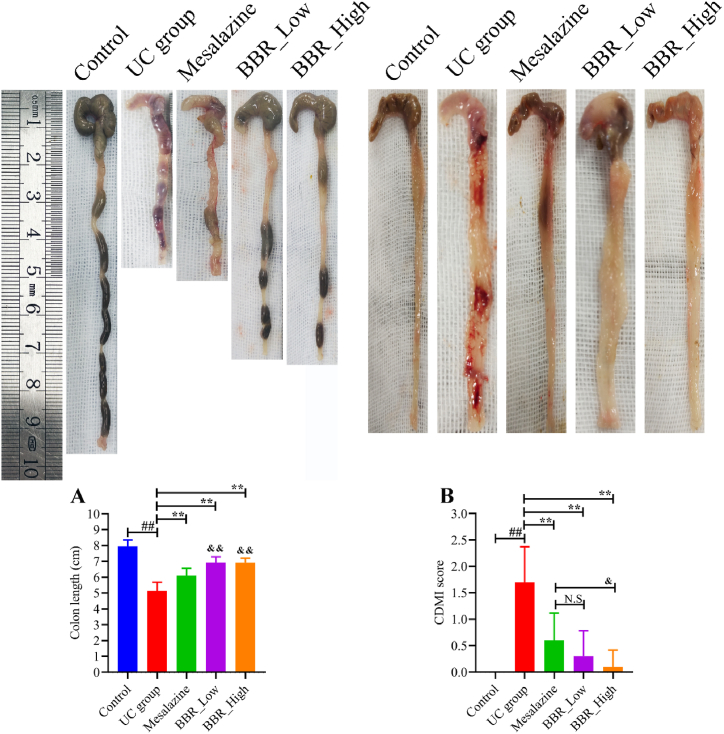
BBR's effect on CMDI values and colon length. A: Measurement of colon length in mice (*n* = 10) from each group; B: Measurement of CMDI values in mice (*n* = 10) from each group. ^&^*P* < 0.05 versus mesalazine group; ∗∗*P* < 0.01 versus UC group; ^&&^*P* < 0.01 versus mesalazine group; ^##^*P* < 0.01 versus control group; N.S: None statistically significant. BBR_Low: Low-dose BBR; BBR_High: High-dose BBR.

A typical characteristic of the DSS-induced UC mice model was colon shortening [27]. The colon length of the UC group was considerably less than that of the control group (Fig. 2A). The colon length was increased significantly following mesalazine and low- and high-dose BBR treatment. The CMDI values in the UC group were considerably higher as compared to the other groups, and the macroscopic histological alterations of the colon mucosa, such as hyperaemia, deem erosion, and ulcer, were also significantly different in the UC group (Fig. 2B). All disease characteristics in the colon were ameliorated owing to mesalazine and BBR treatment (Fig. 2B).

### 3.2 BBR alleviated pathological changes in the colon of UC mice

**Fig. 3:**
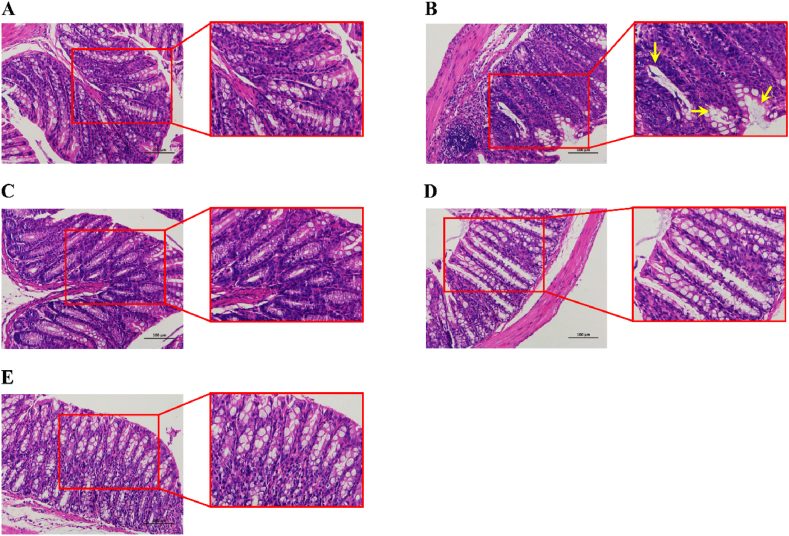
HE staining of colon tissue in mice from each group. Scale bar = 100 μm. A: HE staining of colon tissue in the control group; B: HE staining of colon tissue in the UC group; C: HE staining of colon tissue in the mesalazine group; D: HE staining of colon tissue in BBR with low dose group; E: HE staining of colon tissue in BBR with high dose group.

The HE staining results demonstrated that the colon mucosa in the control group was integral (Fig. 3A). However, there was a loss of colonic mucosal integrity in the UC group, and the crypt location was displaced upward, with irregular and swollen (yellow arrows) crypt shape and significantly reduced goblet cell numbers. Neutrophils and lymphocyte infiltration were seen in the lamina propria, even mostly in the mucosa and submucosa the mucosa and submucosa (Fig. 3B). The mesalazine (Fig. 3C), low-dose BBR (Fig. 3D), and high-dose BBR (Fig. 3E) groups, in contrast with the UC group, showed a reduction in these pathogenic alterations. Furthermore, goblet cell populations in the colon crypts increased following high-dose BBR treatment, while neutrophil and lymphocyte infiltration was considerably reduced (Fig. 3E).

### 3.3 BBR promoted M2 macrophages and suppressed M1 macrophages in the colon of UC mice

**Fig. 4:**
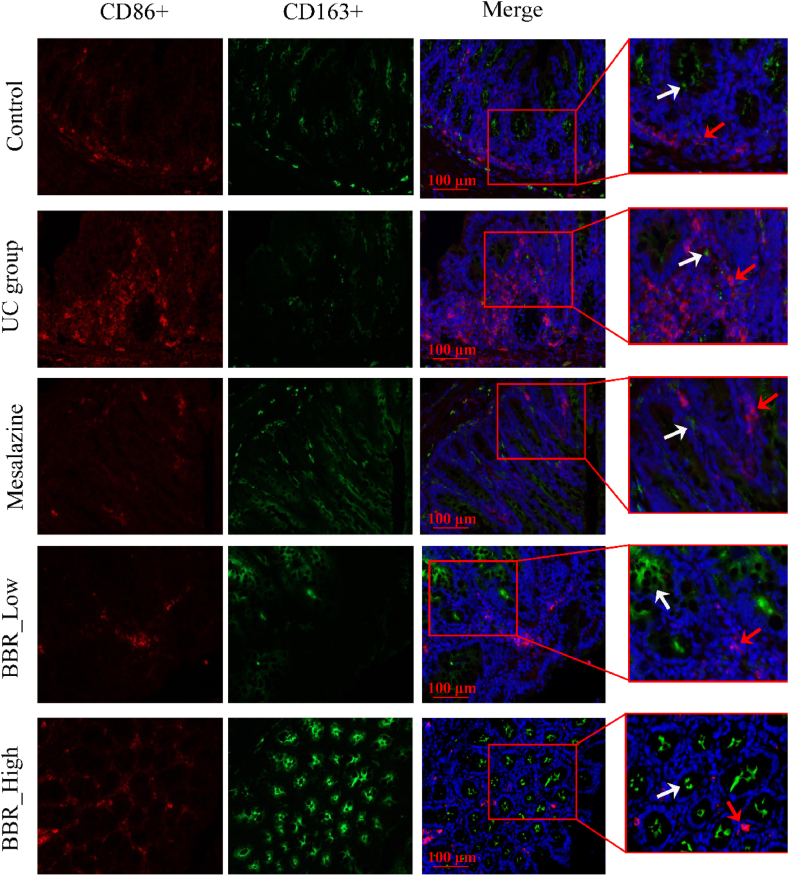
IF staining images of M1/M2 macrophages in colon tissue. Scale bar = 100 μm. M1 macrophages were immunostained by CD86^+^; M2 macrophages were immunostained by CD163+. BBR_Low: Low-dose BBR; BBR_High: High-dose BBR.

CD86^+^ and CD163+ surface markers were used to indicate M1 and M2 macrophages in the colon, respectively. As presented in Fig. 4, CD86^+^ (red arrows) and CD163+ (white arrows) were present in the colon in an appropriate ratio. However, a large number of CD86^+^ markers were accumulated in the colon mucosa in the UC group, which was accompanied by significant CD163+ reduction. After treatment with mesalazine, the CD86^+^ ratio significantly decreased; however, the CD163+ ratio did not significantly increase. Although the CD86^+^ ratio decreased significantly in both the high- and low-dose BBR groups, the CD163+ ratio increased significantly, especially in the high-dose BBR group.

### 3.4 BBR increased M2 macrophages in the abdomen of UC mice

**Fig. 5:**
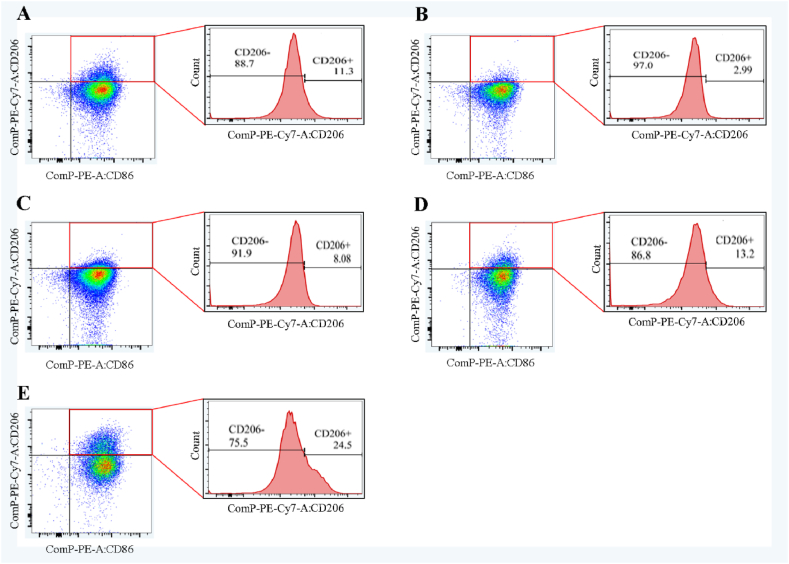
FCM images of M1/M2 macrophages in abdomen from each group mice. A: Percentage of CD11b + F4/80+ CD86^+^ and CD11b^+^ F4/80^+^ CD206^+^ in abdomen of control group; B: Percentage of CD11b + F4/80+ CD86^+^ and CD11b^+^ F4/80^+^ CD206^+^ in abdomen of UC group; C: Percentage of CD11b + F4/80+ CD86^+^ and CD11b^+^ F4/80^+^ CD206^+^ in abdomen of mesalazine group; D: Percentage of CD11b + F4/80+ CD86^+^ and CD11b^+^ F4/80^+^ CD206^+^ in abdomen of BBR with low dose group; E: Percentage of CD11b + F4/80+ CD86^+^ and CD11b^+^ F4/80^+^ CD206^+^ in abdomen of BBR with high dose group.

Previous research found the phenotype and function of peritoneal macrophages were highly correlated with UC based on the activity of reversibility and plasticity [28,29]. The number of CD11b^+^ F4/80^+^ CD206^+^ (M2 macrophages) and CD11b + F4/80+ CD86^+^ (M1 macrophages) cells in the abdomen of the control group were normal, as shown in Fig. 5. However, the number of CD11b + F4/80+ CD86^+^ cells increased in the UC group, which was accompanied by a significant decrease in CD206+ cells (Fig. 5B). After mesalazine administration, the number of CD11b + F4/80+ CD86^+^ cells decreased, while that of CD11b^+^ F4/80^+^ CD206^+^ cells increased slightly (Fig. 5C). In the BBR groups (Fig. 5D and E), the number of CD11b + F4/80+ CD86^+^ cells in the abdomen showed a significant decrease, while that of CD11b^+^ F4/80^+^ CD206^+^ cells did not significantly increase, especially in the high-dose BBR group.

### 3.5 BBR stimulated M2-specific markers in RAW264.7 cells

To evaluate BBR regulation *in vitro*, RAW 264.7 cells were stimulated using LPS (200 ng/mL) and IL-4 (20 ng/mL) to mimic M1-and M2-like macrophages, respectively.

**Fig. 6:**
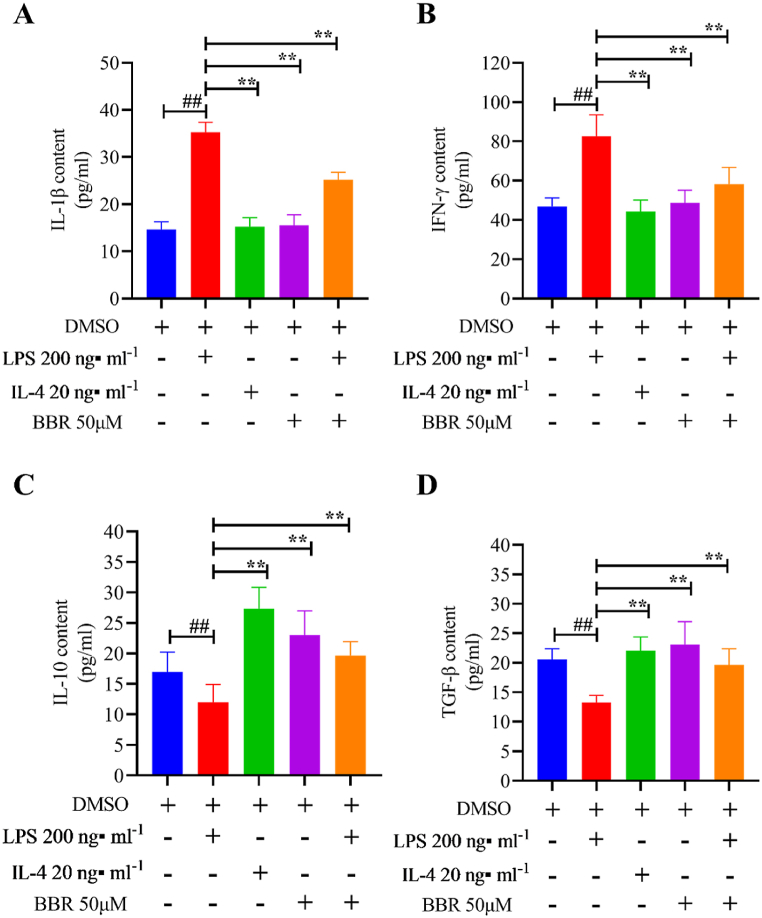
BBR's effect on supernatant indices of RAW 264.7 cells. (A) IL-1β levels in 264.7 cell supernatant; (B) IFN-γ levels in 264.7 cell supernatant; (C) IL-10 levels in 264.7 cell supernatant; (D) TGF-β levels in 264.7 cell supernatant. ∗∗*P* < 0.01 versus the LPS group; ^##^*P* < 0.01 versus DMSO (control) group.

A 50 μM concentration of BBR was chosen as the optimal concentration in RAW 264.7 cells based on our previous study [30]. As shown in Fig. 6, the levels of IL-1β (Fig. 6A) and IFN-γ (Fig. 6B), two M1-specific markers, were considerably elevated in LPS-induced cells than in DMSO-induced cells in the control group. Furthermore, supernatant levels of IL-10 and TGF-β, two M2-specific markers, were considerably lowered after LPS treatment. In contrast, IL-10 (Fig. 6C) and TGF-β (Fig. 6D) levels were substantially higher in BBR- or IL-4-treated cells than in LPS-treated cells. Furthermore, IL-1β and IFN-γ contents decreased significantly in both BBR groups. Therefore, the above findings indicated that BBR suppressed M1-specific markers while promoting M2-specific markers.

### 3.6 BBR upregulated M2 macrophage gene expression in RAW264.7 cells

**Fig. 7:**
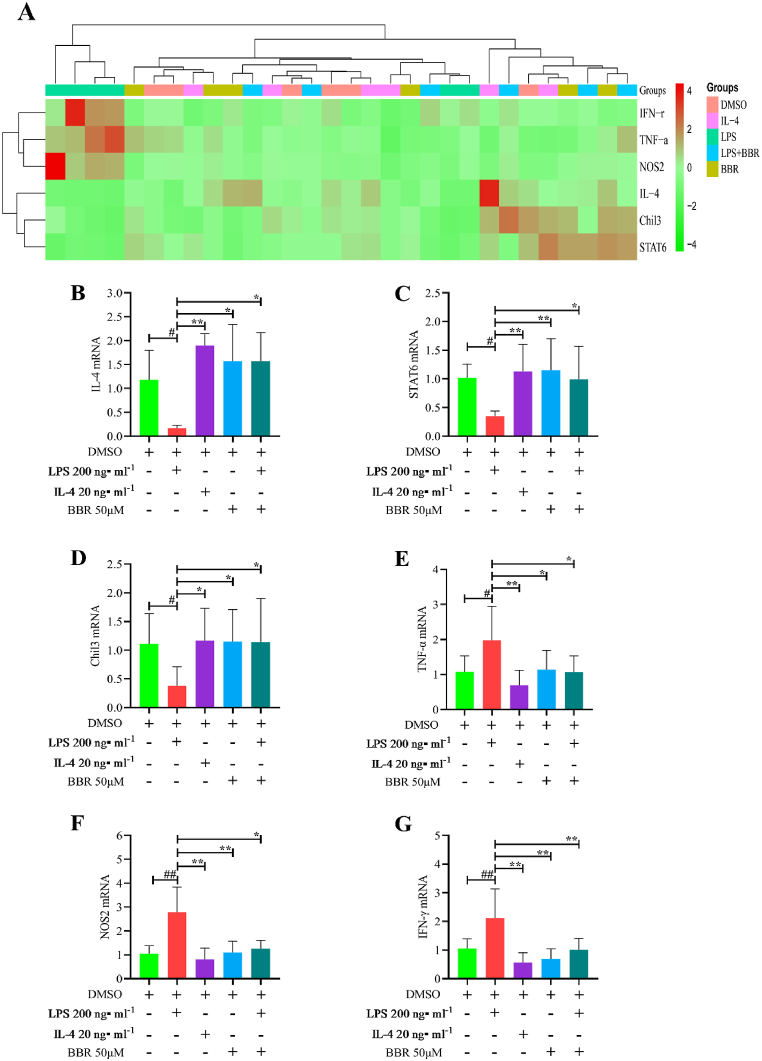
BBR's effect on the mRNA expression of RAW 264.7 cells. A: Heatmap of mRNA expression in each group; B: mRNA expressions of IL-4; C: mRNA expressions of STAT6; D: mRNA expressions of Chil3; E: mRNA expressions of TNF-α; F: mRNA expressions of NOS2; G: mRNA expressions of IFN-γ. ∗*P* < 0.05 versus LPS group; ∗∗*P* < 0.01 versus LPS group; ^#^*P* < 0.05 versus DMSO group; ^##^*P* < 0.01 versus DMSO group.

The mRNA expression of M1-and M2-like macrophages following BBR intervention was assessed using qRT-PCR. Fig. 7A displays the heatmap results. The mRNA expression of M2-like macrophages, including IL-4 (Fig. 7B), STAT6 (Fig. 7C) and Chil3 (Fig. 7D), showed a significant decrease in the LPS-induced groups. However, TNF-α (Fig. 7E), NOS2 (Fig. 7F), and IFN-γ (Fig. 7G) mRNA expression increased significantly in the LPS-induced groups. Compared to the LPS groups, IL-4 and BBR substantially increased M2-like macrophage mRNA expression. In addition, compared to the LPS groups, BBR inhibited TNF-α, NOS2, and IFN-γ expression. Overall, our findings suggest that BBR increases M2-like macrophage gene expression level while decreasing M1-like macrophage gene expression level.

### 3.7 BBR activated the IL-4-STAT6 signalling pathway *in vivo*

**Fig. 8:**
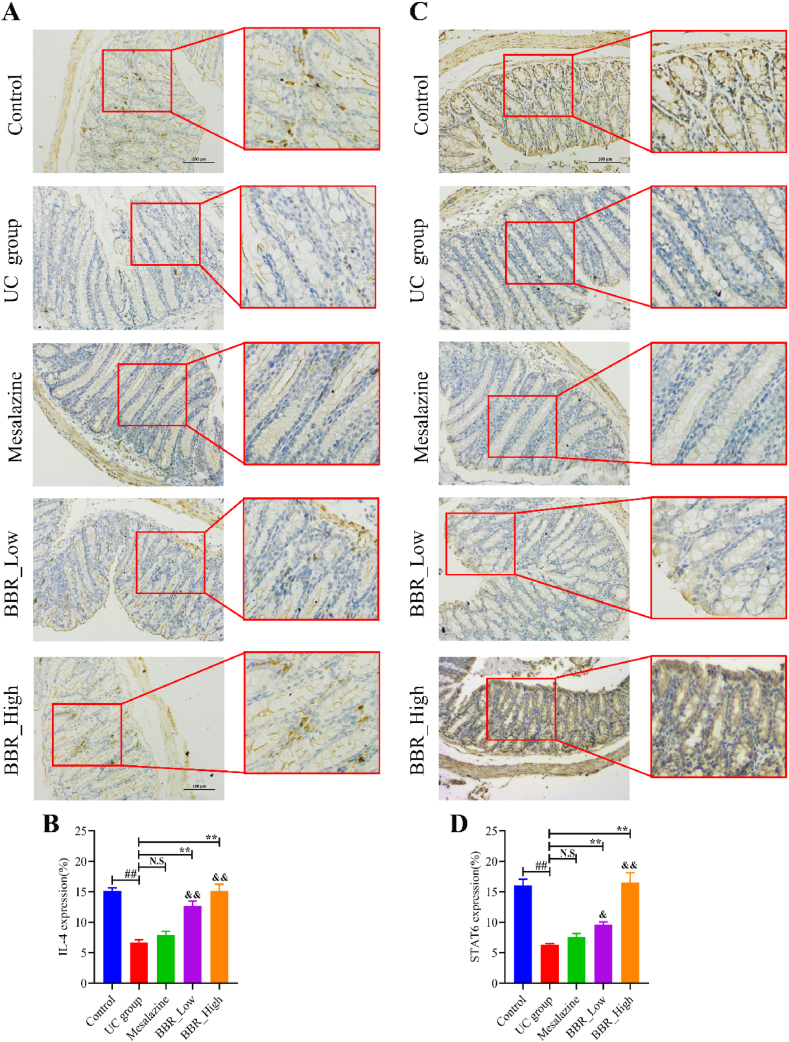
Results of IHC staining in mice from each group. A: IHC staining plot of IL-4 in colon tissues; B: IL-4 in colon tissue as per quantitative analysis results; C: IHC staining plot of STAT6 in colon tissue; D: STAT6 in colon tissue as per quantitative analysis results. ^&^*P* < 0.05 versus mesalazine group; ^&&^*P* < 0.01 versus mesalazine group; N.S: None statistically significant; ∗∗*P* < 0.01 versus UC group; ^##^*P* < 0.01 versus control group. BBR_High: High-dose BBR; BBR_Low: Low-dose BBR.

The IL-4-STAT6 signalling pathway has been reported to be a prerequisite for M2 macrophage polarisation [31,32]. Therefore, the effect of BBR on the IL-4-STAT6 signalling pathway in the colon of UC mice was examined. As shown in Fig. 8, IHC analysis revealed that IL-4 was positive and expressed at high levels in the control group (Fig. 8A and B). However, IL-4 was negative in the UC group. After mesalazine treatment, IL-4 expression showed no significant increase (Fig. 8A and B). However, after BBR treatment, IL-4 expression increased significantly compared with the UC group (Fig. 8A and B). Correspondingly, the positivity of STAT6 was also decreased in the UC group (Fig. 8C and D). STAT6 expression increased considerably compared to the UC group after treatment with BBR, particularly at high doses (Fig. 8C and D).

**Fig. 9:**
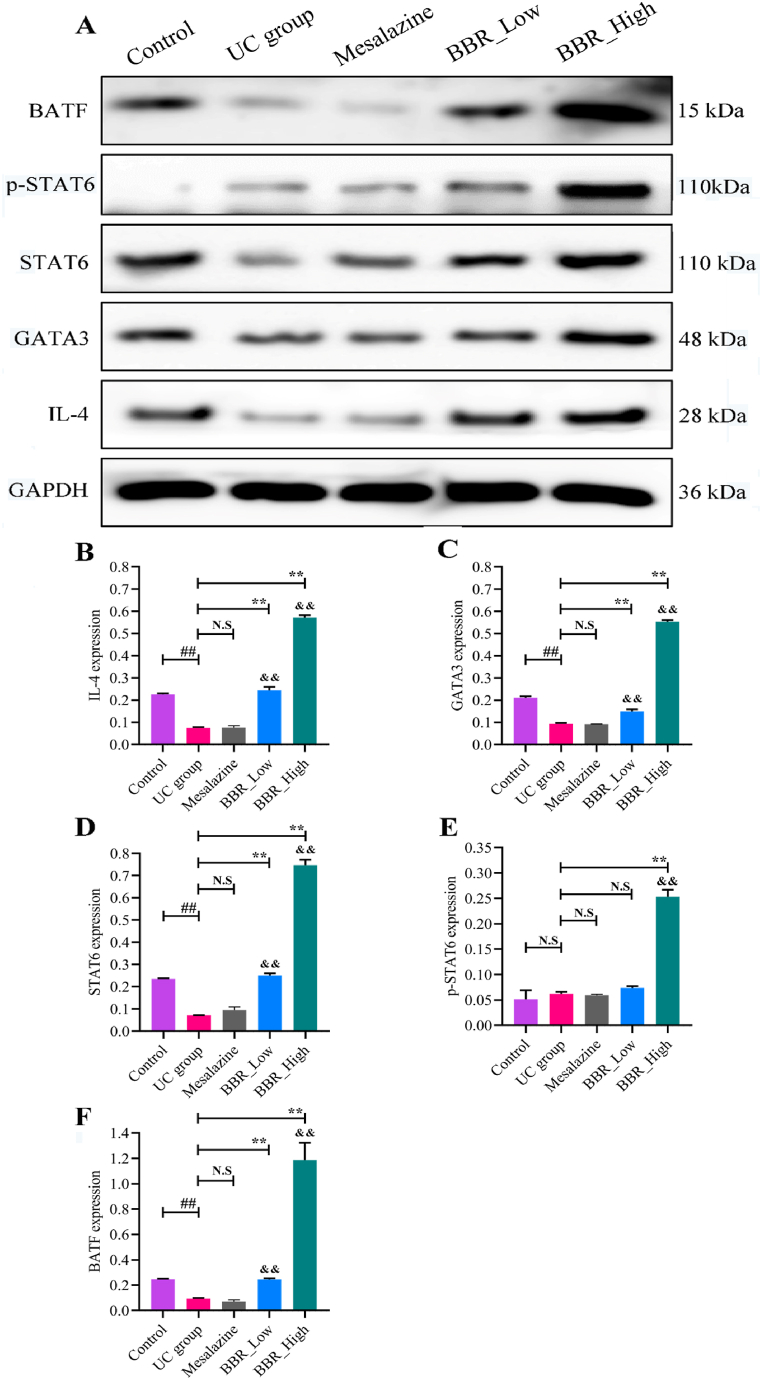
Expression of the protein in mice from each group assessed using WB analysis. A: IL-4, STAT6, GATA3, BATF, and p-STAT6 (Tyr641) WB images. B: Relative expression of IL-4 protein in colon tissue; C: Relative expression of GATA3 protein in colon tissue; D: Relative expression of STAT6 protein in colon tissue; E: Relative expression of p-STAT6 protein in colon tissue; F: Relative expression of BATF protein in colon tissue. ∗∗*P* < 0.01 versus UC group; ^##^*P* < 0.01 versus control group; ^&&^*P* < 0.01 versus mesalazine group; N.S: None statistically significant. BBR_High: High-dose BBR; BBR_Low: Low-dose BBR.

Was BBR truly activating the IL-4-STAT6 signalling pathway? To answer this question, WB analyses were conducted to evaluate the protein expression of IL-4, GATA3 (a direct downstream target of IL-4), p-STAT6, STAT6, and BATF (a direct STAT6-dependent transcription factor) [33] in UC mice. In Fig. 9A, the control group expressed relatively more IL-4, GATA3, STAT6, and BATF than the UC group; however, p-STAT6 expression was unchanged in both groups. Moreover, following mesalazine treatment, the expression of these proteins did not significantly increase. Contrarily, the expression of IL-4 (Fig. 9B), GATA3 (Fig. 9C), STAT6 (Fig. 9D), p-STAT6 (Fig. 9E), and BATF (Fig. 9F) were significantly upregulated in the BBR groups. These proteins showed significant differences between the BBR low-and high-dose groups. WB analyses further confirmed that BBR induced M2 macrophage polarisation in UC mice by activating the IL-4-STAT6 signalling pathway. WB-derived original images of colon tissue are listed in Supplement 2.

### 3.8 BBR promoted the IL-4-STAT6 signalling pathway *in vitro*

**Fig. 10:**
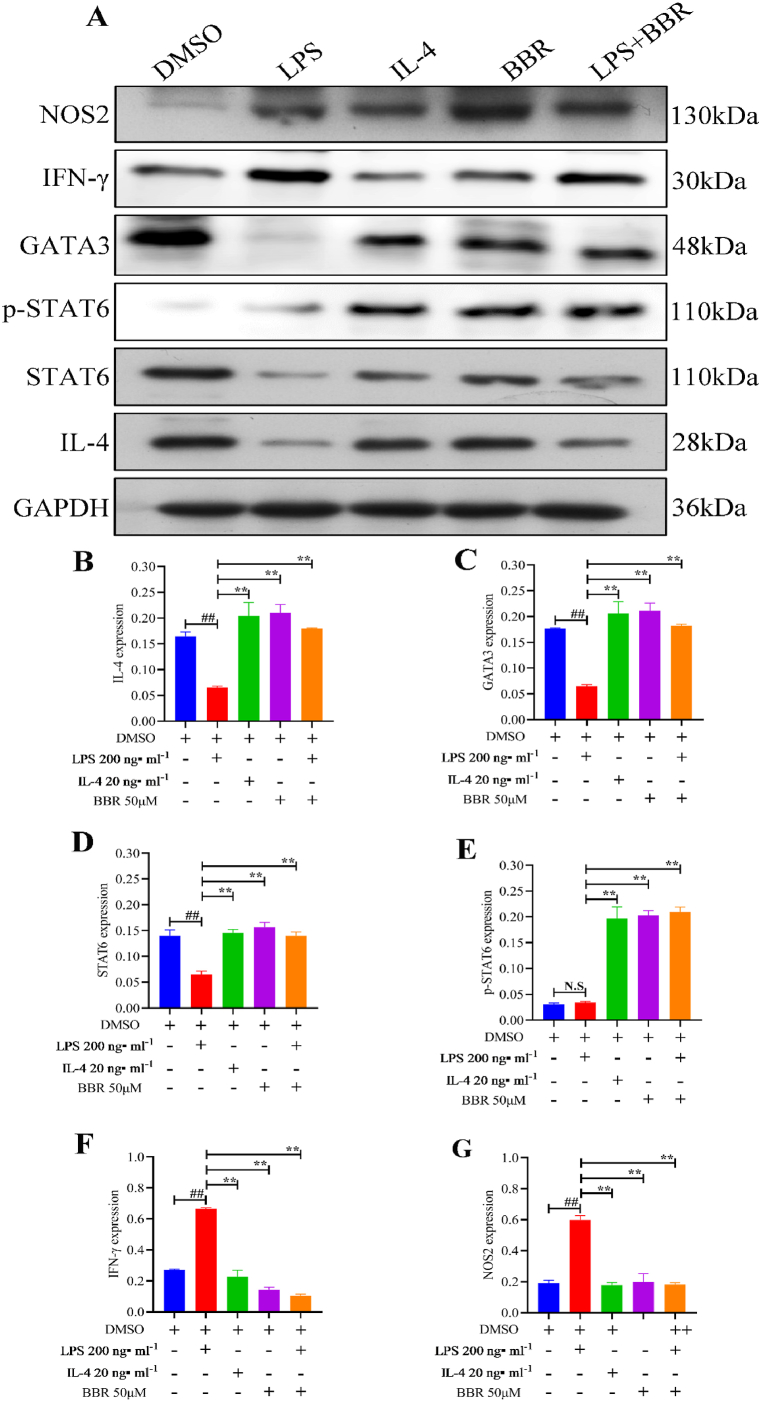
Protein expression in RAW264.7 cells assessed using WB analysis. A: WB images of IL-4, GATA3, STAT6, p-STAT6 (Tyr641), IFN-γ, and NOS2. B: Relative IL-4 expression of the protein in RAW264.7 cells; C: Relative expression of GATA3 protein in RAW264.7 cells; D: Relative expression of STAT6 protein in RAW264.7 cells; E: Relative expression of p-STAT6 protein in RAW264.7 cells; F: Relative expression of IFN-γ protein in RAW264.7 cells; G: Relative expression of NOS2 protein in RAW264.7 cells; N.S: None statistically significant; ∗∗*P* < 0.01 versus LPS group; ^##^*P* < 0.01 versus DMSO group.

BBR's effect on the IL-4-STAT6 signalling pathway was further verified using *in vitro* experiments. As presented in Fig. 10A, LPS exposure significantly attenuated the expression of GATA3, IL-4, and STAT6. However, the expression of p-STAT6 was not significantly affected by LPS. Additionally, IFN-γ and NOS2, which are M1-like macrophage markers, showed significantly increased expression on LPS induction. In comparison to the LPS groups, IL-4 and BBR substantially stimulated IL-4 (Fig. 10B), GATA3 (Fig. 10C), and STAT6 (Fig. 10D). In addition, p-STAT6 (Fig. 10E) expression was significantly upregulated by IL-4 and BBR, whereas IFN-γ (Fig. 10F) and NOS2 (Fig. 10G) were suppressed on IL-4 and BBR intervention. These results indicated that BBR stimulated the IL-4-STAT6 signalling pathway *in vitro*. WB original images of Raw 264.7 cells are listed in Supplement 3.

## 4 Discussion

According to the findings of this study, M1 macrophage is the primary macrophage type mainly distributed in the DSS-induced inflammatory colon. M2 macrophages in the colon of UC mice were deprived owing to the increasing M1-like macrophages. Furthermore, BBR increased the efficacy of UC treatment by suppressing M1 macrophages and enhancing M2 macrophages to exhibit anti-inflammatory activity *in vivo* and *in vitro*. Furthermore, BBR activated the IL-4-STAT6 signalling pathway to induce the polarisation of M2 macrophages, which was extremely crucial for the alleviation of inflammation.

Macrophages are the first line of defence in the innate immune response and are primarily produced from monocytes in the blood [34]. Macrophages are widespread throughout tissues, mucosal surfaces, and bodily cavities, such as the gastrointestinal tract, lung dust cells, and skin Langerhans cells [35]. *In vivo*, macrophages play a crucial role in tissue formation and internal environment homeostasis. They undergo timely polarisation in response to particular stimuli, and their subtypes may accurately regulate and respond to numerous stimuli, which might impact inflammation or disease progression [8]. The classical activation of macrophages (M1) and the alternative activation of macrophages (M2) are the two well-characterised outcomes of macrophage polarisation based on different stimuli [8,9]. M1 macrophages are mainly induced by LPS, TNF-α, granulocyte-macrophage colony-stimulating factors, and IFN-γ [36,37], which express high levels of G-protein coupled receptor 18, CD38, CD68, CD80, CD86, MHC-II molecules, and formyl peptide receptor 2 [38,39]. M1 macrophages can exacerbate the pro-inflammatory response by producing pro-inflammatory cytokines, including CCL15, CCL20, CXCL8-11, IL-1β, CXCL13, IL-12, IL-18, TNF-α IL-23, and IL-6. Parallelly, M2 macrophages are primarily induced by macrophage colony-stimulating factors, TGF-β, IL-4, and IL-13. M2 macrophages are characterised by their anti-inflammatory activities and high anti-inflammatory cytokine levels, including TGF-β, IL-13, IL-10, and IL-13.

M1-M2 macrophage homeostasis is critical because imbalances can lead to the onset and progression of various diseases. Various studies have demonstrated that the polarisation of M1 macrophages was enhanced, which contributed to serious colon inflammation and deteriorates UC progression [14,40]. Conversely, the conversion from M1 to M2 macrophages or M2 macrophage promotion in UC is most likely to alleviate colon inflammation and contribute to tissue repair [13,41]. IL-4, a key inducer of M2 macrophage polarisation and T cell differentiation, also has tissue-repair, anti-inflammatory, and M1 macrophage polarisation properties [9]. IL-4 forms a complex by binding to the cytokine-binding receptor IL-4Rα and further recruits the secondary receptor IL-2Rγc or IL-13Rα1 to form a functional receptor complex [42]. In addition, IL-4 induces downstream signalling via IL-4Rα, with the Y residue in the intracellular IL-4Rα domain acting as a docking site for the SH domain of the intracellular signalling molecule to activate STAT6 [43]. Additionally, p-STAT6, a macrophage transcriptional repressor, combines with macrophage lineage-determining transcription factors (such as RUNX family transcription factor 1 and activation protein 1) and increases the transcription of genes regulating M2 polarisation [44,45]. IL-4-induced STAT6 activation significantly inhibits NLRP3, IL-1β, and IL-18 expression [46]. Therefore, the IL-4-initiated and STAT6-transcripted signalling pathways dominate M2 macrophage polarisation.

In inflammatory diseases, BBR, a natural isoquinoline alkaloid, has been shown to induce M2 macrophage polarisation [30,47]. This study reports that BBR inhibited M1-polarised macrophages and promoted M2-polarised macrophages in UC. The alleviation of inflammatory lesions in the colon was accompanied by the increased proportion of M2 macrophages in the colon and abdomen in UC mice after BBR treatment, whereas M1 macrophages were suppressed significantly on BBR treatment. Moreover, *in vitro* experiments revealed a significant decrease in the supernatant biochemical biomarkers of M1 macrophages, including IL-1β and IFN-γ. BBR treatment, on the other hand, significantly increased IL-10 and TGF-β levels. Additionally, our findings showed that BBR activated the IL-4-STAT6 signalling pathway during M2 macrophage polarisation. BBR increased the expression level of GATA3 and BATF as well as IL-4 and STAT6. In addition, p- STAT6 was activated by BBR. These results indicated that BBR promoted M2 macrophage polarisation through the IL-4-STAT6 signalling pathway in treating UC. But our study has some limitations. (1) reverse validation is not performed; (2) other mechanisms may contribute to BBR's therapeutic effect on UC; and (3) BBR's therapeutic effect on UC needs to be further validated in human clinical trials.

## 5 Conclusion

BBR was effective in treating DSS-induced UC, supporting its anti-inflammatory role in the treatment of UC. BBR's anti-inflammatory effects were strongly related to the inhibition of M1 macrophages and the stimulation of M2 macrophages, which activated the IL-4-STAT6 signalling pathway.

## Author contribution statement

Kai Xiong: Conceived and designed the experiments; Performed the experiments; Analysed and interpreted the data; Wrote the paper. Jia Deng, Wenting Hu, Tinghui Yue, Xinglin Zeng: Performed the experiments; Wrote the paper. Tao Yang, Tianbao Xiao: Conceived and designed the experiments; Contributed reagents, materials, analysis tools or data.

## Funding statement

Tao Yang was supported by National Natural Science Foundation of China [82260936]; National key R & D project [2018YFC1704500]; special funding for Ph.D of Guizhou University of Traditional Chinese Medicine [GYZYYFY- BS-2022(02)].

## Data availability statement

Data included in article/supplementary material/referenced in article.

## Declaration of interest's statement

The authors declare that they have no known competing financial interests or personal relationships that could have appeared to influence the work reported in this paper.
