# Supplementary material for: Berberine promotes M2 macrophage polarisation through the IL-4-STAT6 signalling pathway in ulcerative colitis treatment
Source: Heliyon. 2023 Mar 1;9(3):e14176. doi: 10.1016/j.heliyon.2023.e14176 (PMC10009548; doi:10.1016/j.heliyon.2023.e14176)
Supplement: Multimedia component 1 [file mmc1.pdf]

Western Blot original images of colon tissue

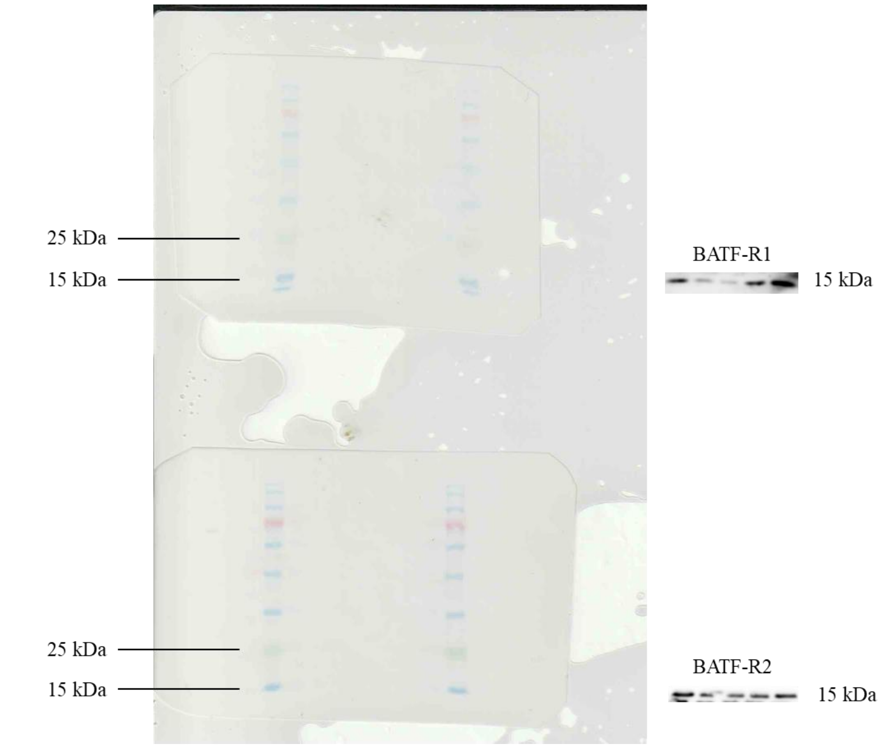

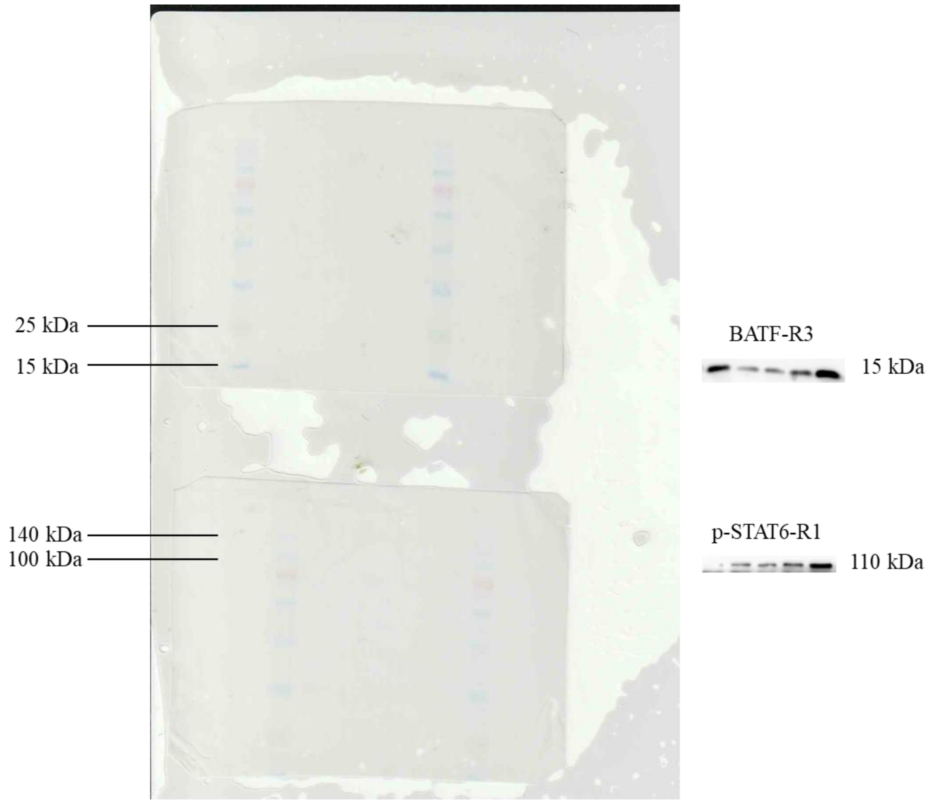

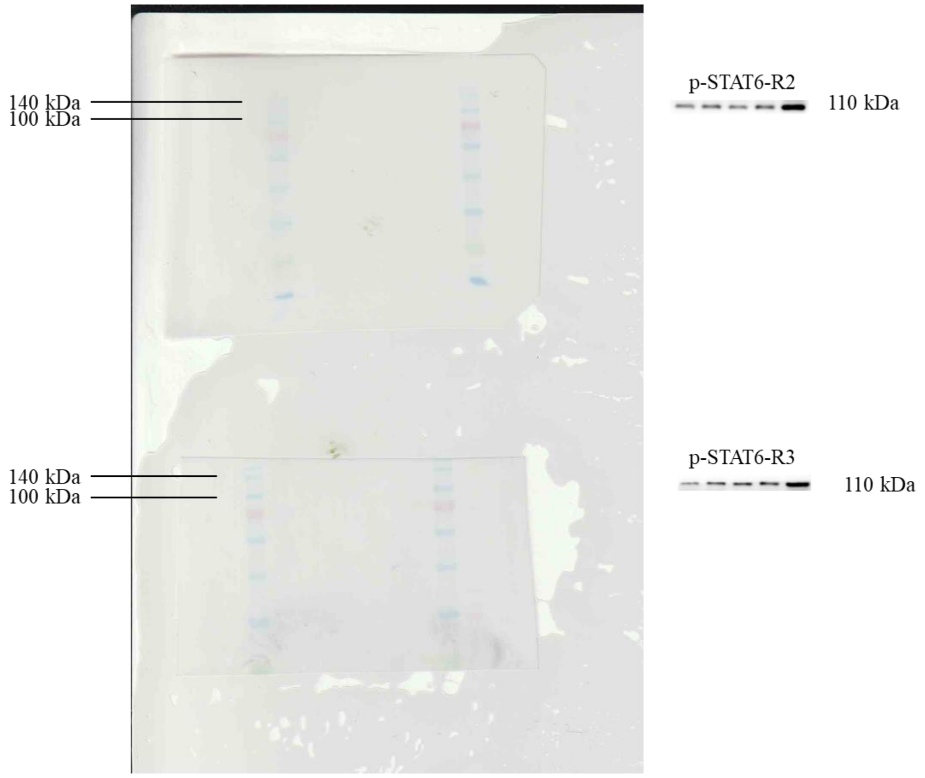
140 kDa
100 kDa
p-STAT6-R2
110 kDa
140 kDa
100 kDa
p-STAT6-R3
110 kDa

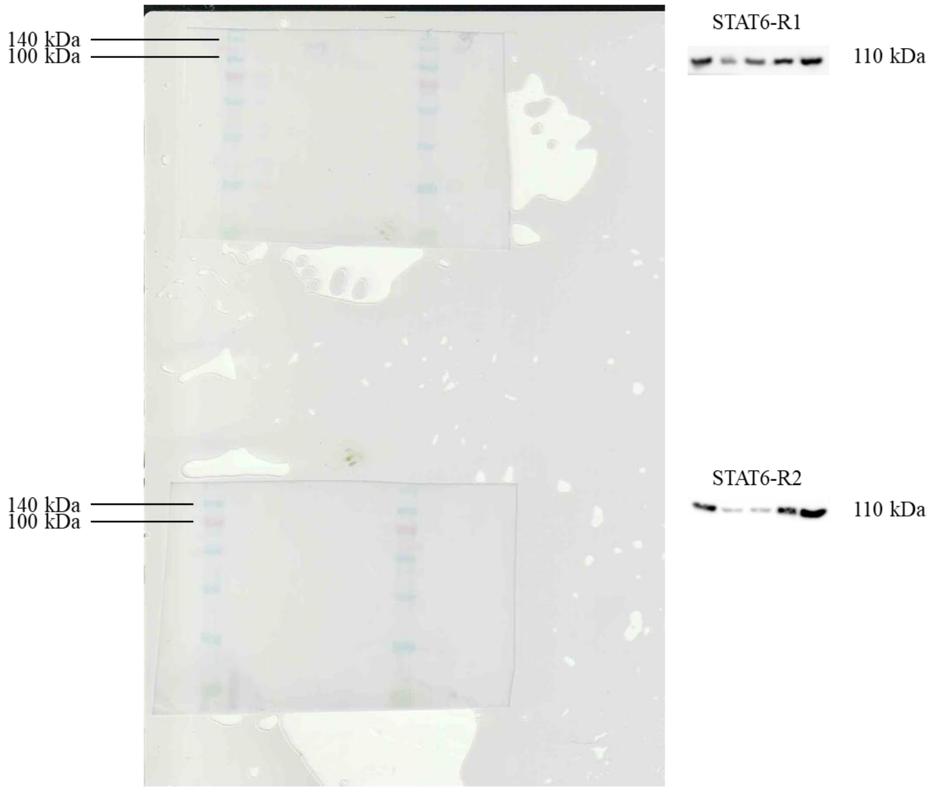
140 kDa
100 kDa
STAT6-R1
110 kDa
140 kDa
100 kDa
STAT6-R2
110 kDa

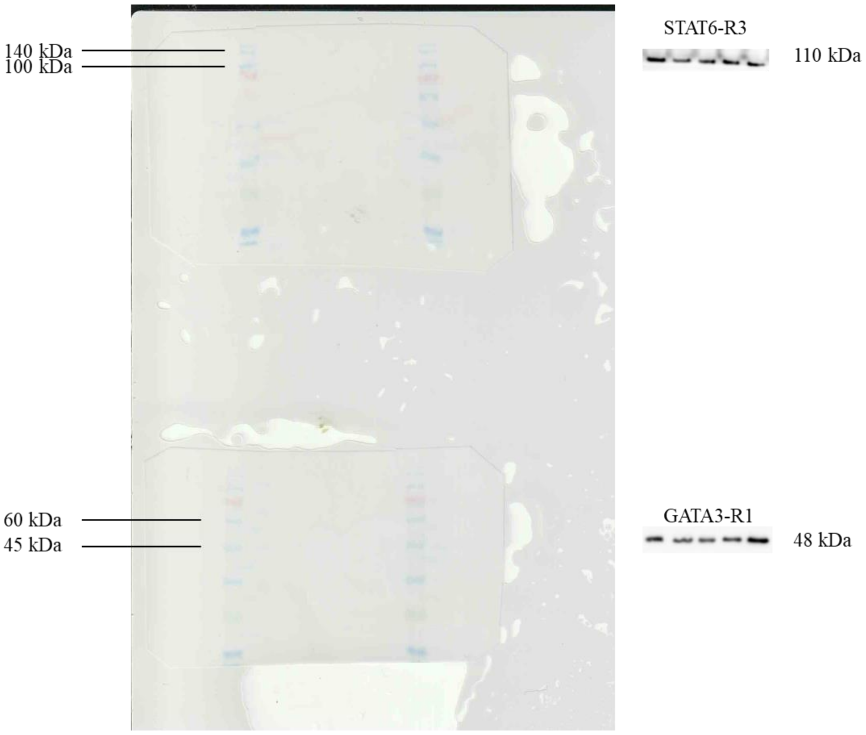
140 kDa
100 kDa
60 kDa
45 kDa
STAT6-R3
110 kDa
GATA3-R1
48 kDa

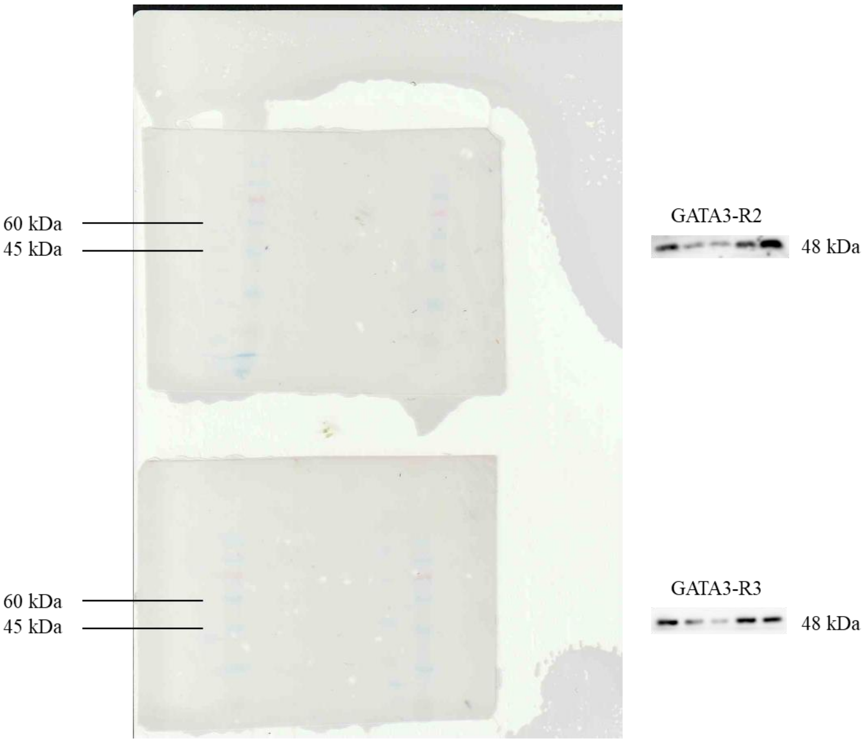
60 kDa
45 kDa
60 kDa
45 kDa
GATA3-R2
48 kDa
GATA3-R3
48 kDa

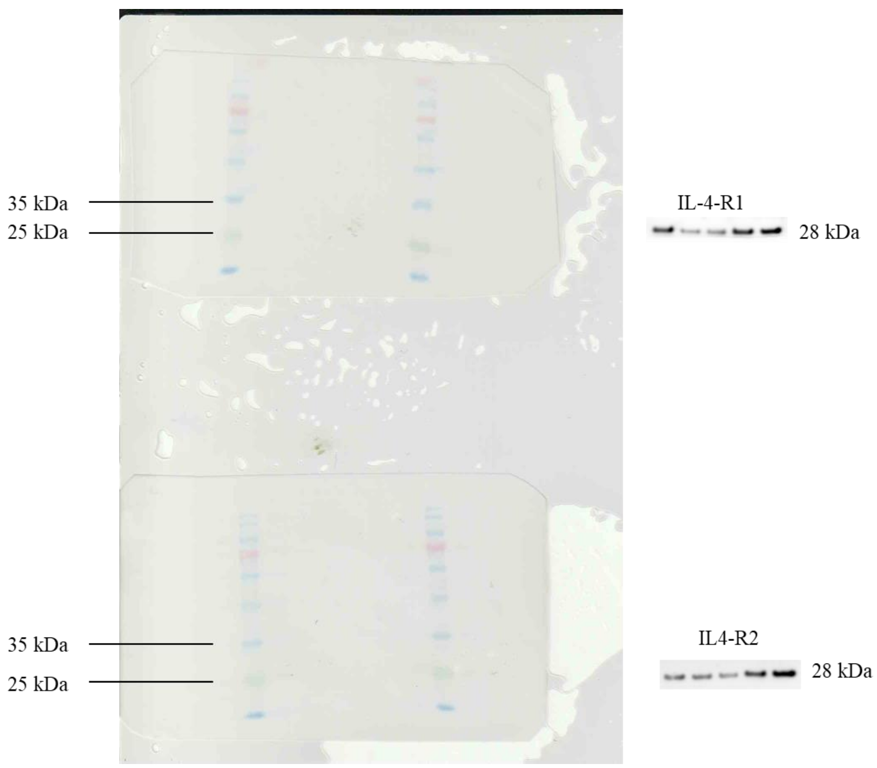
35 kDa
25 kDa
IL-4-R1
28 kDa
35 kDa
25 kDa
IL4-R2
28 kDa

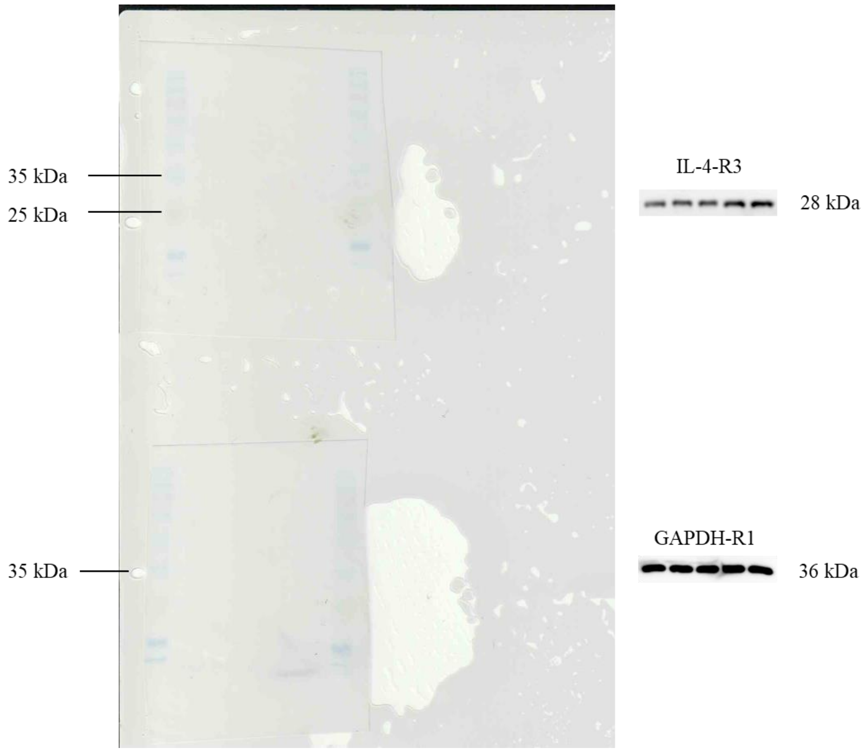
35 kDa
25 kDa
IL-4-R3
28 kDa
35 kDa
GAPDH-R1
36 kDa

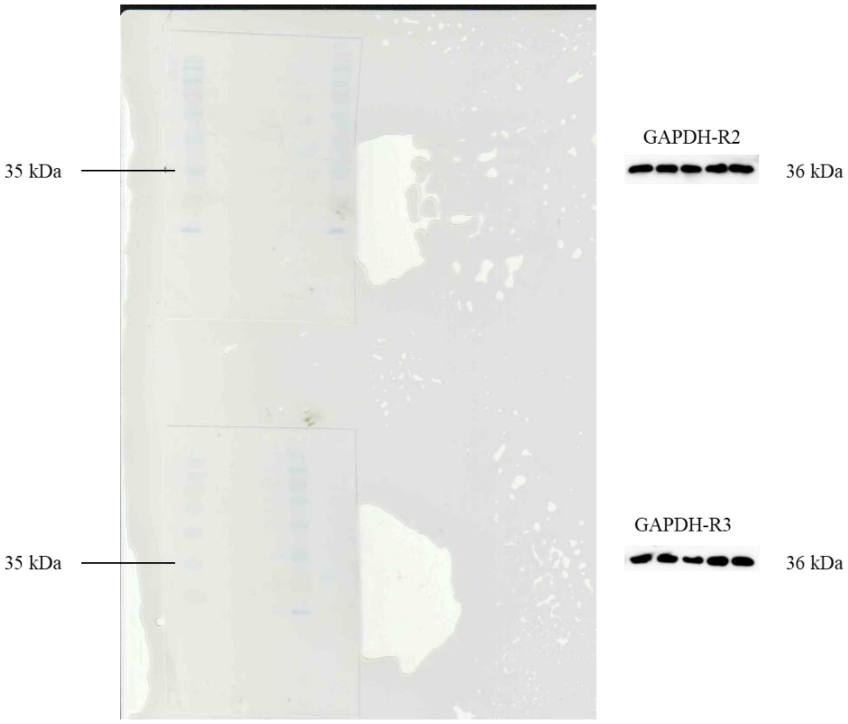

Western Blot Protocol

（1） Sample Preparation (Total protein)

1.1 For suspension cells: Centrifuge at 3000 rpm, 4°C for 4minutes to collect cells, add RIPA buffer. (250μL RIPA buffer/10^6 cells（Protease and phosphatase inhibitors should be added before use）.

1.2 For adherent cells

1.2.1 Place the cell culture dish on ice and wash the cells three times with ice-cold PBS.

1.2.2 Aspirate the PBS, then add ice-cold RIPA buffer (Protease and phosphatase inhibitors should be added before use).

1.2.3 Scrape adherent cells off the dish, then gently transfer the cell suspension into a 1.5 mL microcentrifuge tube.

1.3 Maintain constant agitation for 30 min in ice bath, use pipette to mix it up.

1.4 Centrifuge at 12,000 rpm, 4 ℃ for 10 min. Gently remove the tubes from the centrifuge and place on ice, tranfer the supernatant to a fresh tube.

（2） Preparation of lysate from tissues

2.1 Wash the sample with cold PBS for twice or three times， then homogenize with an electric homogenizer. Volumes of lysis buffer must be determined in relation to the amount of tissue.

2.2 Maintain constant agitation for 30 min in ice bath, use pipette to mix it up.

2.3 Centrifuge at 12,000 rpm ,4 ℃ for 10 min. Gently aspirate the supernatant and place in a fresh tube kept on ice.

（3）Histone extraction protocol for western blot

3.1 Prepare the reagents： Dissolve the reagents at room temperature, then mix them up and put them on ice.

The Nuclear protein and cytoplasmic protein extraction reagent A and B should be mixed up with PMSF until its final concentration comes to 1mM.

3.2 Adherent cells： Wash the cells three times with ice-cold PBS or EDTA. Centrifuge and collect the cells, discard the supernatant.

3.3 Suspension cells： Wash the cells three times with ice-cold PBS or EDTA. Centrifuge and collect the cells, discard the supernatant.

3.4 Fresh Tissue**：**

Cut the tissue into very small pieces as much as possible. Mix the appropriate amount of cytoplasmic protein extract A and B at a ratio of 20:1 (e.g., add 10 microliter of cytoplasmic protein extract B to 200 microliter of cytoplasmic protein extract A). Prepare a tissue homogenate with a final concentration of PMSF of 1 mM. Add 200μL of lysis buffer per 60mg tissue for machine homogenization. The homogenization should be performed in an ice bath or 4 ℃. Place in an ice bath for 15 minutes. After centrifugation at 2000g , 4°C for 5 minutes, the supernatant was transferred to a precooled EP tube, which was part of the cytoplasmic protein extracted.(Never touch the sediment when supernatant.) At this stage, some plasma proteins were still not extracted, and the remaining precipitation was again extracted by the method of cell precipitation.

3.5 Add 200μL of cytoplasmic protein extraction reagent A with PMSF into each 20μL of cell precipitate.

3.6 Vortex at the highest speed for 5s, let the cell precipitates completely suspended and dispersed;(Vortex time can be extended if cell precipitation were not completely suspended and dispersed).

3.7 Maintain constant agitation for 10-15 min in ice bath.

3.8 Add 10μL Cytoplasmic protein extraction reagent B (add PMSF before use). Vortex at the highest speed for 1s. Maintain constant agitation for 1 min in ice bath.

3.9 Vortex at the highest speed for 5s， Centrifuge at 12,000-16,000g, 4 ℃. for 5 min.

3.10 Aspirate the supernatant (plasma protein) and place in a fresh tube kept on ice.

3.11 Aspirate the supernatant. Add 50μL nuclear protein extraction reagent B(add PMSF before use).

3.12 Vortex at the highest speed for 15-30s. Maintain constant agitation in ice bath. Vortex at the highest speed for 15-30s in between 1-2 minutes. (Total:30min).

3.13 Centrifuge at 12,000-16,000g, 4 ℃ for 10 min.

3.14 Tranfer the supernatant to a fresh tube kept on ice. The supernatant is nuclear protein, you can immediately use or keep in -80°C fridge.

（4）Determination

If the protein concentration needs to be measured, take some undenatured protein solution and measured by the BCA protein concentration measurement kit. The specific method is referred to the kit instructions

（5）Denature the protein

Add 5* reduced protein loading buffer to the protein solution at a ratio of 4:1, denature it in a boiling water bath for 15 minutes, and stored at -20°C for later use

（6）SDS-PAGE

6.1 Washing glass plates.

6.2 Preparing and loading the gel.

6.2.1 After the glass panel naturally dried, a concave glass panel and a flat glass panel shall be formed into a pair, which shall be put into the gel-making device, inserted into the inclined glass panel to fix the glass panel, and check whether the bottom is aligned to avoid gel leakage;

6.2.2 Different concentrations of separation gel were prepared according to the experimental requirements, and mixed evenly immediately after adding TEMED, and the separation gel was poured to an appropriate height. A comb could be used to test the height before pouring, and the comb teeth should be about 5-8mm away from the liquid level of separation gel. Then add the pure water to the top layer of the separation gel slowly and evenly until it is filled. After about 30 min, pour the water from the upper layer of the separation glue and drain the remaining water with absorbent paper.

6.2.3 Prepare 5% concentrated gel according to the formula. After adding TEMED, mix well immediately, and then the gel can be filled. Fill the remaining space with the concentrate and insert the comb into the concentrate.

Make sure there are no bubbles under the comb.

6.2.4 After stacking gel has polymerized, remove comb carefully and begin the electrophoresis.

6.3 Add enough electrophoresis solution for sample electrophoresis (Marker: 5 μl, Sample: 10 μl), Concentrated gel voltage is 75V, and separation gel uses 120V. The electrophoresis was stopped when the bottom of bromophenol blue was about 1cm.

（7） Transferring the protein from the gel to the membrane

7.1 Prepare 6 pieces of 7×9cm filter paper and a PVDF (0.45um) membrane of moderate size. The PVDF membrane should be activated by methanol for 2min before use

7.2 In the basin with transfer buffer, place the clip for transfer film, two sponge pads, a glass rod, filter paper and activated PVDF film;

7.3 Open the clip with white on the left and black on the right. Add a sponge and three layers of filter paper on each side.

7.4 Carefully peel off the separation glue and put it on the filter paper. Put the PVDF film on the filter paper. Cover three pieces of filter paper on the film and remove the bubbles. Finally, cover with another spong.

7.5 Transfer condition **(**wet transfer) 300mA for 30 min, during the transfer process, the film transfer equipment is placed in ice water to cool down

(8) Antibody staining

8.1 Put the transferred membrane into the TBST incubation tank for a quick Wash, Block the membrane for 30minutes at room temperature by blocking buffer (5% milk,).

8.2 Incubate the membrane with appropriate dilutions of primary antibody. We recommend overnight incubation at 4 ℃.

8.3 Recycle the primary antibody then wash the membrane for three washes with TBST, 5 min each.

8.4 Incubate the membrane with the recommended dilution (1:5000) of conjugated secondary antibody in blocking buffer at room temperature for 30min.

8.5 Wash the film three times with TBST, 5minutes each time.

(9) Chemiluminescence

Acquire image using darkroom development techniques for chemiluminescence, Perform ECL as described by the manufacturer, add ECL reagents for 1-2min at RT and capture WB image using various durations of exposure.

(10) Image analysis

Scan the film, Organize and desaturate by using Photoshop. Use Alpha processing system to analyze the optical density value of the target band.

(11) WB result and analysis
