# Supplementary material for: Berberine promotes M2 macrophage polarisation through the IL-4-STAT6 signalling pathway in ulcerative colitis treatment
Source: Heliyon. 2023 Mar 1;9(3):e14176. doi: 10.1016/j.heliyon.2023.e14176 (PMC10009548; doi:10.1016/j.heliyon.2023.e14176)
Supplement: Multimedia component 2 [file mmc2.pdf]

Western Blot original images of Raw 264.7 cells

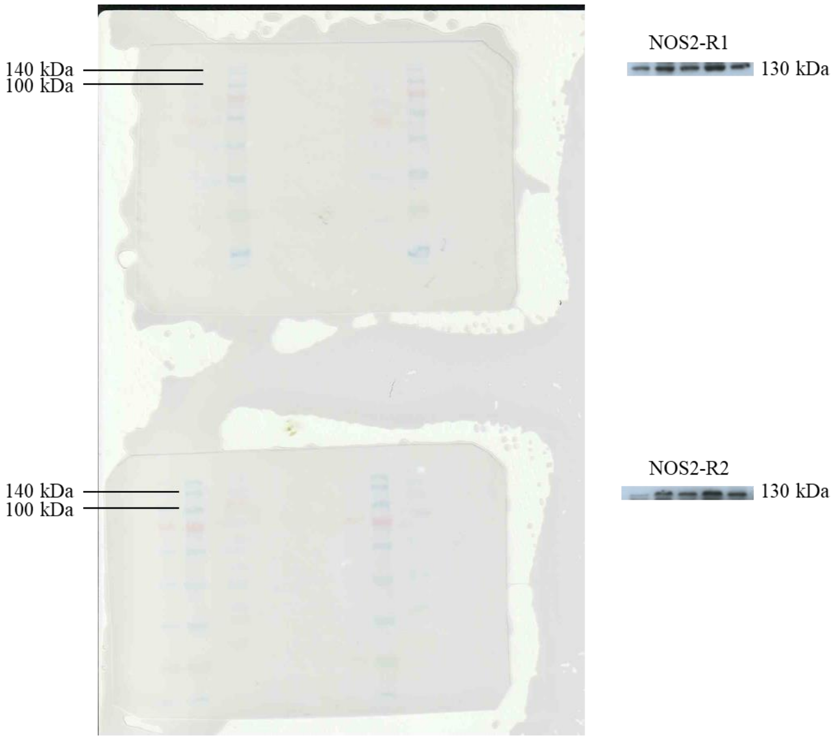

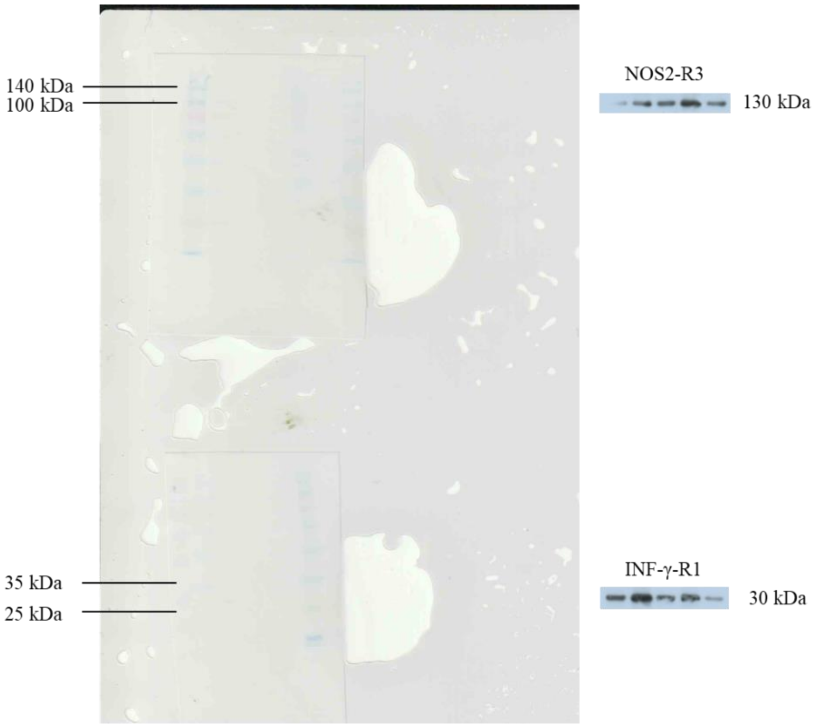

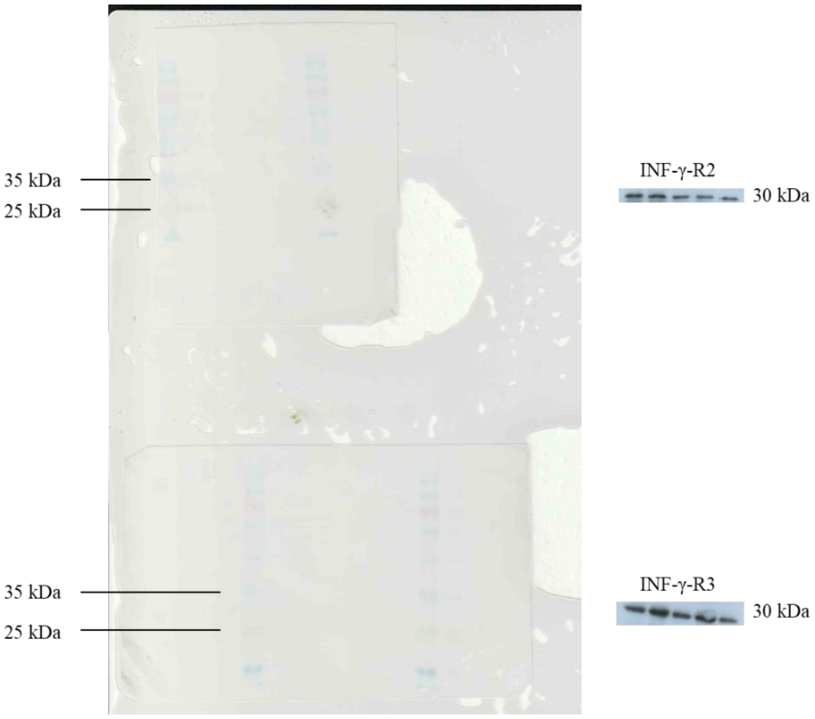
35 kDa
25 kDa
INF-γ-R2
30 kDa
35 kDa
25 kDa
INF-γ-R3
30 kDa

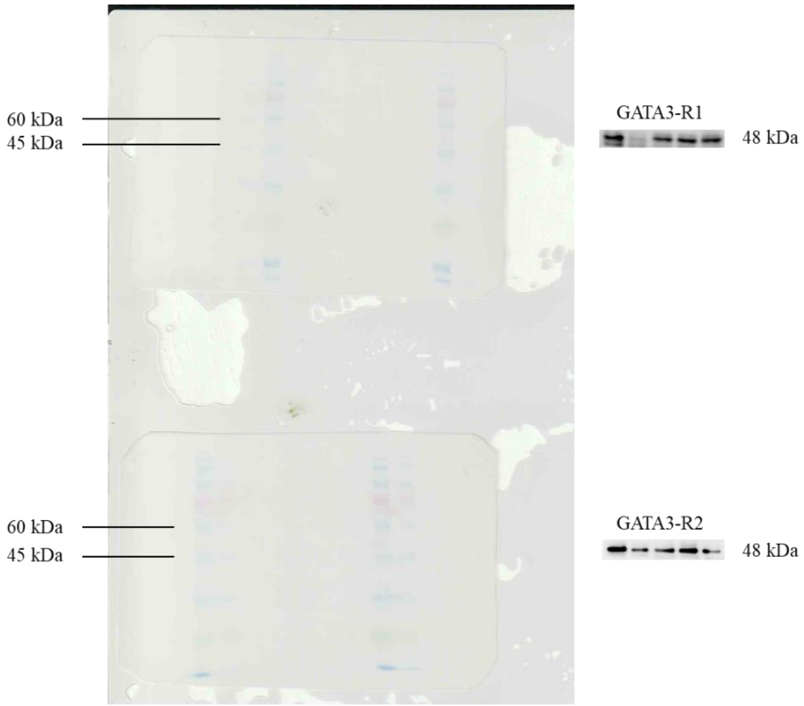
60 kDa
45 kDa
GATA3-R1
48 kDa
60 kDa
45 kDa
GATA3-R2
48 kDa

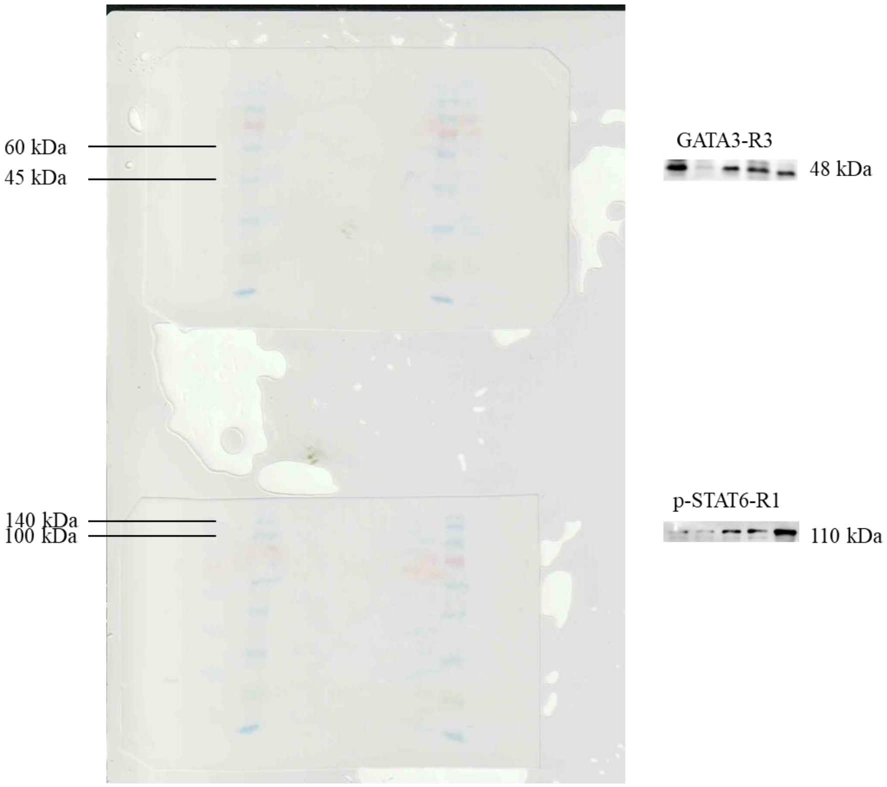
60 kDa
45 kDa
140 kDa
100 kDa
GATA3-R3
48 kDa
p-STAT6-R1
110 kDa

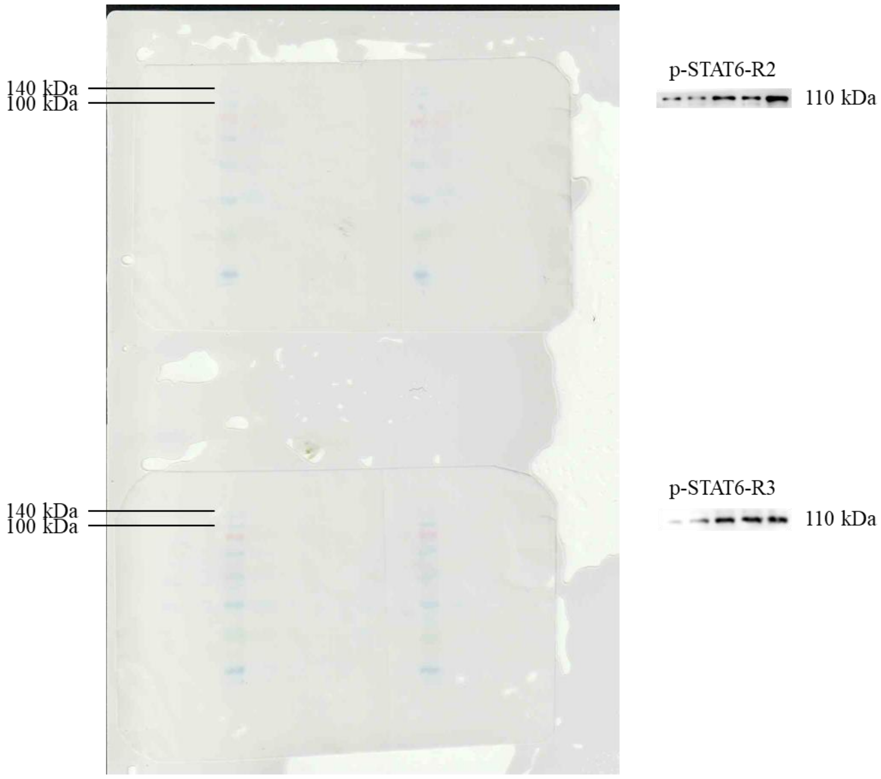
140 kDa
100 kDa
140 kDa
100 kDa
p-STAT6-R2
110 kDa
p-STAT6-R3
110 kDa

140 kDa
100 kDa

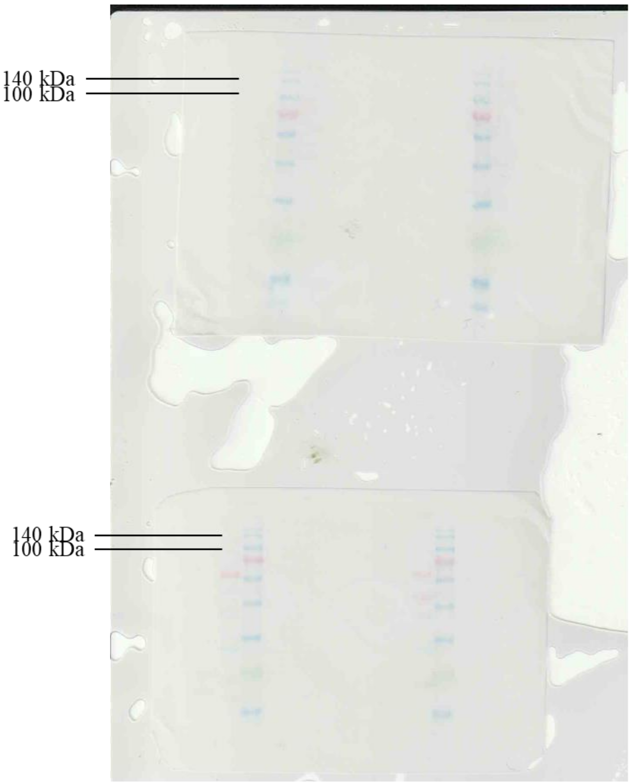

STAT6-R1

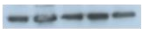

110 kDa

140 kDa
100 kDa

STAT6-R2

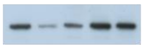

110 kDa

140 kDa
100 kDa

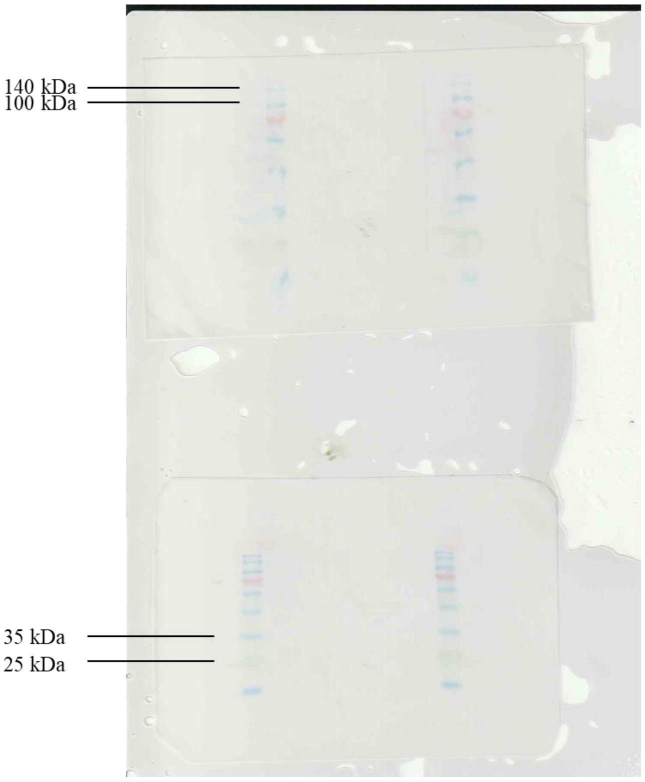

STAT6-R3

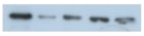

110 kDa

35 kDa
25 kDa

IL-4-R1

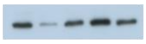

28 kDa

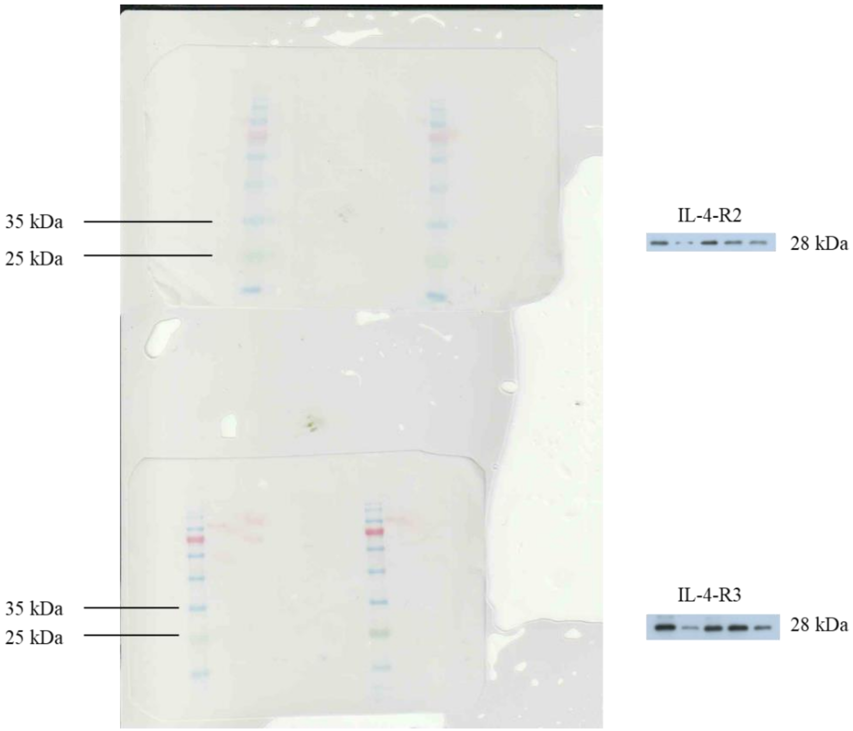
35 kDa
25 kDa
IL-4-R2
28 kDa
35 kDa
25 kDa
IL-4-R3
28 kDa

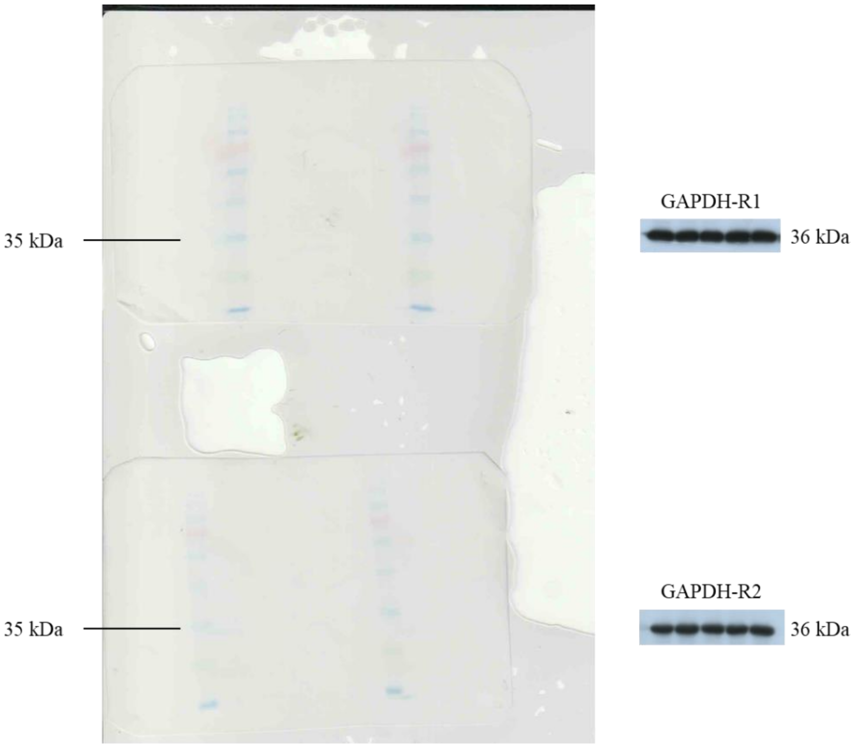
35 kDa
GAPDH-R1
36 kDa
35 kDa
GAPDH-R2
36 kDa

35 kDa

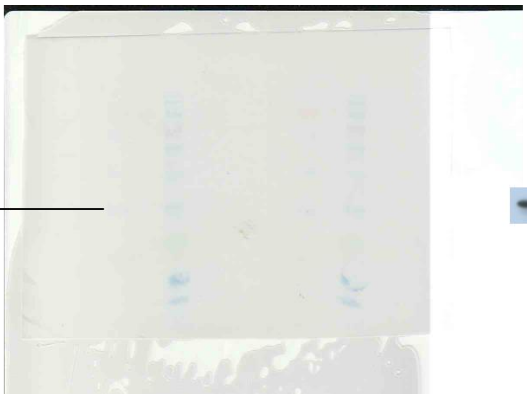

GAPDH-R3

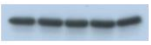

36 kDa
